# The synergy between food and agri-food suppliers, and the restaurant sector in the World Heritage City of Córdoba (Spain)

**DOI:** 10.1186/s42779-022-00126-7

**Published:** 2022-03-14

**Authors:** Cinthia Rolim de Albuquerque Meneguel, Ricardo David Hernández-Rojas, Manuel Rivera Mateos

**Affiliations:** 1 Department of Tourism, Federal Institute of Education, Science and Technology of São Paulo - Campus Cubatão, Rua Maria Cristina 50, Cubatão, São Paulo 11533-160 Brazil; 2 Department Agricultural Economics, Sociology, and Policy, Faculty of Economics and Business Sciences, University of Córdoba, Plaza de Puerta Nueva S/N, 14002 Córdoba, Spain; 3 Tourism Analysis and Prospective Center, University of Córdoba, Pedro López de Alba Building, Calle Alfonso XIII, 13, 14071 Córdoba, Spain

**Keywords:** Local food product, Traditional food, Sustainable food, Gastronomy tourism, Food tourism, Food heritage, Food and culture, Food supplier, Catering sector, Hospitality supply chain

## Abstract

The tourist-catering subsector plays an important role in the consolidation and attraction of tourism products and services offered. This research aims to ascertain the importance of synergies, and proactive collaborative and co-operational relations between agri-food suppliers and the restaurants. This is a qualitative, exploratory and descriptive methodology, data source triangulation, information processing using NVIVO12 software. This study identifying that the city of Córdoba offers a variety of gastronomic products and services, and that the supply sector is semi-structured and still in need of improvements of quality, safety and having a wider commercial network. Despite the COVID-19 pandemic, suppliers have reinvented themselves and created new market opportunities to benefit from rapid growth in some sectors; furthermore, they are in a position to offer restaurants a competitive advantage in post-pandemic recovery.

## Introduction

For many tourist destinations, local gastronomy and tourist catering services have become a strategic factor to providing visitors with a positive experience that is often distinctive and strongly linked to a geographical identity [35, 52]. Thus, themes rooted in distinctive *terroir* characteristics are being especially proposed in gastronomy tourism initiatives at this time of reorganization of activity after 2020 will belong to the local [8, 34, 75].

The food culture of an ethnic group is influenced by the beliefs, behaviours, religions, values and social norms of the community that come from the accumulation of local culture, a legacy of authenticity from the previous generation [82]. Thus, traditional cuisine gives it individual and unique characteristics, associated with various cultural elements present in this process that are part of an identity recognition, which can give it value and become a precious attraction for the tourist cultural activity [7, 75, 94]. In this way, valuing intangible characteristics of the territory, such as traditions, stories, memories, techniques, habits that are sharing the authenticity of the agri-food technique and cuisine, highlighting the sociocultural appreciation of the resources of rural territories perceived as a set of material and immaterial elements linked to production and the agricultural environment inserted in the perception of a collective territorial heritage [15, 89].

Food has its intrinsic value and its ethical and symbolic values that are translated through taste [71]. Thus, tourists perceive, in effect, the restaurant subsector as an important attribute of the destination. Therefore, the quality of the gastronomy and service, along with the physical surroundings, can all produce a profound sensory and cultural experience of significant impact and level of satisfaction, whose several case studies have demonstrated, as in New Zeland [5], Ireland [22, 23], India [4], China [15], Italy [28], Colombia [84] and Indonesia [59].

For these reasons, the past twenty years have seen tourist catering, as part of the larger hotel and catering industry, become the focus of an increasing amount of scientific research [21],however, few empirical studies have been carried out in this regard [85]. In any case, the restaurant subsector is contributing significantly to the economic development of many destinations and to the increase in employment—given that it is a very labour-intensive subsector—to territorial revitalization and the strengthening of the brand image of tourist destinations, favouring, likewise, the valorisation, enjoyment and recognition of native gastronomy and agri-food products, culinary recipes and traditional dishes that also make up the hallmarks of cultural and heritage identity of the territories.

Gastronomy and the tourist-catering subsector in the province of Córdoba play an important role in the region’s economic and sociocultural development. The city’s unique cultural melting pot has been the subject of study in numerous areas: segmentation of food market visitors [79], artificial neural networks [71], general characteristics and trends [72]*,* defining the tourism catering service [29], Designations of Origin [43, 65, 66, 68, 69]; the rights of users and obligations of companies [30],wine tourism [40, 103], tourist satisfaction levels [80]; traditional gastronomy [41]; the nutritional value of traditional dishes [73], a gastronomic tourism observatory [38], olive oil tourism [64, 99]; restaurant client profile [37, 39], gastronomy tourism motivations [6, 57], agri-food products in gourmet shops [42]; gastronomic routes [48, 67]; industrial gastronomic tourism [63, 100, 101] and gastronomic tourism versus culinary tourism [101, 102]*.*

Since the end of the global economic crisis of 2008–2012, there has been a boom in tourism in Córdoba, consistently ranking among the 15 most competitive Spanish city destinations [98]. But, since March 2020, Córdoba has been profoundly affected by the suspension of tourist activity decreed by the Spanish government in response to the coronavirus SARS-COV2 (COVID-19) pandemic. The situation has worsened in the second quarter, as the tourist high-season coincides with the all but total closure of hotels and restaurants. Estimates put losses at over €65 million, and a fall of over 80% in visitors. Such statistics led the Spanish government to implement a packet of measures aimed at minimizing the economic impact of COVID-19 and helping to maintain the tourist sector afloat through credit lines, loans, reductions of 50% in social security payments, and the renegotiation of commercial rents. Despite these measures, the sector still faces numerous challenges before it becomes sustainable once more.

Córdoba has high-quality gastronomic products and services, a strong local identity and historic-cultural tradition, which includes Roman, Arab, Jewish, Christian and Latin-American elements; all of these in turn are further mixed with the innovative elements of avant-garde cuisine, attributes on ethnic food identity formation [46]. The city has improved its national and international position as a destination for city breaks and cultural tourism [65, 66] and this cannot be understood without referring to the high quality of the city’s restaurants.

Therefore, this study aims to understand the synergy between food and agri-food suppliers, and the restaurant sector in the World Heritage City of Córdoba (Spain). We have identified prior research into how relations between food suppliers and/or agri-food producers and the restaurants are viewed. However, the focus of these studies is on the experience of chefs, or restaurant management, rather than on the relations between supplier and purchaser, which is the focus of this study [50, 74, 86–88, 92]. Corroborating with Chopra and Meindl [17], who have shown that a more holistic and integrated perspective is called for, one that takes into account intangible aspects related to the flow of information, communication, integration and synergy between suppliers and purchasers (restaurants), among other analytical variables.

Despite the evidently attractive nature of the subject, this is the first study into the aforementioned synergies, and their implications for business management in the city’s restaurant sector. Thus, this innovative research is an initiative of theoretical and empirical contribution that, through its results, presents a significant diagnosis and the importance of the positive synergies and proactive relations between those who play a role in local tourism, and in the efficient operation of the tourist value chain.

## Conceptualizing the research

Andalusia is the second largest of the country’s 17 Autonomous Regions, and one of the most important and competitive of Spain’s tourist destinations, with tourism accounting for 12.5% of the region’s GDP, and around 12% of its employment [24, 95].

Córdoba is also one of Andalusia’s eight provinces. It has a wide range of territorial and landscape resources, which has led to very varied agricultural production, with a great diversity of high-quality agri-food produce, and numerous, complementary tourist products. These regional quality certificates result in important social and economic benefits that aid in promoting rural areas, as well as providing extra income and support to combat unfair competition, while at the same time raising restaurant clients’ awareness of this problem [97]. These official designations boost the gastronomic tourism sector around specific food produce and recipes [22], to such an extent that governments and diverse institutions recognize the value of gastronomy as a seal of local authenticity and identity, thus protecting the products, promoting ecological awareness and the healthy and sustainable use of agri-food products, and stimulating inter-culturalism [10, 59, 83].

The tastes of Córdoba are intrinsically linked to a number of ingredients that are expressly recognized by Designations of Origin. These certificates not only guarantee the quality of the ingredients, but also serve to certify the quality and branding of those restaurants that use them, as well as the tourist imaginary of a cultural and gastronomic destination [15, 93].

This should always be borne in mind in a city such as Córdoba, where the service sector, which includes tourism and catering, is the most important single sector in the city’s economy [76]. However, the destination still has to face the challenge of offering new tourist products and experiences that should solve some of the city’s structural problems, including short average stays in the city, and a strongly seasonal demand; as mentioned, a further challenge is now the recuperation of tourism in the post-COVID-19 era.

Córdoba is one of the cities that best represents the idea of cultural tourism in Spain. While it is home to key heritage that can be visited, such as the UNESCO declared World Heritage Sites (Mosque-Cathedral 1984, Historic City Centre 1994, The Festival of Cordoban Patios 2012, Medina Azahara archaeological complex 2018), and also benefits from other, wider-ranging recognitions, such as that of Flamenco 2010, and the Mediterranean Diet 2013, these are insufficient to solve the problems of structure and economic sustainability in the city’s tourism.

Taking this as a starting point, and with the clear need to extend and enrich the city’s supply of tourism products and services, gastronomy and restaurants of great gastronomic interest can play a role in opening new opportunities and perspectives, thus improving competitiveness and aiding the city’s overall economy [15, 62]. In Córdoba’s case, it is the city’s culture that attracts a clear majority of visitors, although the second reason is its gastronomy [6, 58]. One of the main strengths of Córdoba’s gastronomy is the quality of its typical, traditional dishes [72]*.* Such motivations efficiently contribute to making tourists’ experience more profound and identity-linked, increasing their knowledge of the city and the likelihood, they will return.

In addition to the city’s vast historical, architectural and cultural heritage, tourists are also highly satisfied with Cordoban gastronomy. Ranging from traditional taverns in the courtyards of typical Andalusian houses to tourist restaurants offering Mediterranean food, it also includes more creative. Furthermore, the city has 14 restaurants with some kind of Michelin recognition [31]. In 2014, the city was recognized as Ibero-American Gastronomic Capital, an award that recognizes the quality of its food heritage [3].

The Spanish agri-food sector receives great support from public–private sector actions that help business development. The Government of Spain, through its Ministry of Agriculture, Fisheries, and Food (2020), has launched an institutional publicity campaign using the hashtags #Explora, #Saborea, #Disfruta, #Comparte los Alimentos de España [70]. The campaign promotes and recognizes the quality and variety of Spanish food produce, as well as the diversity of agri-food cultures around the country, and links gastronomy to the image of Spain in a number of contexts, like the fine work of chefs and restaurants and tourism.

## Agri-food suppliers

Management of the agri-food supply chain involves a large number of agents at all stages from the very earth the product comes from, through the production system, the processing, transformation, and its distribution, sale, to its inclusion in the dishes offered by restaurants [9, 11, 90], without forgetting the inverse processes that seek to ensure the sustainability, quality and competitiveness of said agri-food production [27], aim to ensure sustainability, quality and productivity of agri-food production [32].

Among these implications, the Globally Important Agricultural Heritage Systems–GIAHS, understand that food security and biodiversity conservation are fundamental and closely interconnected challenges to which traditional and sustainable agri-food production systems, evolving system of human communities that accumulation of experience over generations, in an intricate relationship with their territory, cultural, agricultural landscape or biophysical environment [25].

Therefore, the opportunities and challenges that the agri-food and restaurant sectors face should be considered in relation to the socio-economic, environmental, cultural, tourism and nutritional impacts that result from the processes and synergies, which can be more or less unequal or functional, existing between the various phases of production and commercialization [2]. All of these phases require prior planning to ensure the product’s organoleptic quality, freshness, naturalness and conservation as a perishable good at until final consumption [33].

Restaurants of a certain gastronomic quality and more oriented towards tourists should understand that the final process of marketing and sales is an important operational activity that is a key factor in their success [51]. This process should be addressed via a well-defined strategy of competitiveness and sustainability that produces benefit, increases profitability and achieves the client’s satisfaction and loyalty [84, 88].

A greater integration and positive synergies between the external resources offered by suppliers and the internal resources of restaurants who seek competitive positioning [49, 104], such fundamental integration is based on achieving mutual commercial goals [33]. A number of studies [32, 81, 104] have shown that this functional integration with suppliers strengthens and intensifies restaurants’ sustainable management.

A restaurant management precisely demonstrated the profits such establishments make through the integration of local suppliers’ resources, and the maintenance of fluid relations, benefitting the local economy, promoting the possibility of purchasing smaller quantities of fresher, safer, higher quality produce, as well as ensuring the satisfaction of the client and their level of trust and knowledge regarding the origin of the food they consume, and how it is processed and produced [45, 81]. There is also a notable improvement in the management of the business itself, as well as in the favourable inter-relations between all those involved in the tourism and agri-food value chain [106], and final market competitiveness [2], leading to greater cost reduction, minimizing logistic chain failures [18, 50] and identifying coming changes to supply chain trends [45].

Murphy and Smith [74] also recognized the importance of building fluid relations with restaurant suppliers as a way of understanding the flow of gastronomic goods and services and obtaining first-hand information regarding the demand for new dishes and menus, culinary and technological novelties or solutions to operational problems [32]. Mistakes made by suppliers in product choice and distribution may directly or indirectly affect the operational management of catering companies [16], thus the exchange of information and lessons learnt can prevent negative inertia in business management.

## Local agri-food products and their integration in the gastronomic offer of restaurants

For a long time, gastronomy, modern cuisine, and especially new fast-food trends have become disconnected from local agri-food produce and its roots [82]. This disconnection has also occurred with respect to our territorial cultures, what "we really are" and our origins to the extent that we buy and taste food that is increasingly influenced and mediated by circumstances external to our territorial environment of residence, both of a socioeconomic and socio-political nature, in addition to the inertia of globalization [26].

The recent change in food-connected behaviour and habits is related to an increasing interest in the certified quality and geographical origin of agri-food, as well as in the differentiating agro-ecological and geographical criteria used in their production and elaboration. These changes are taking place in a context in which tourist restaurants need to reinvent and position themselves as one leisure experience among many others [54]. Current needs for new groups who are increasingly well organized and closely linked to the local producers themselves [13].

The local product, understood as belonging to a defined geographical region, with specific territorial qualities that condition the natural characteristics of its agri-food production, is also the result of a specific historical-cultural environment where techniques, knowledge, cultural experiences, traditions and have had an impact on the socio-economic development of the area and its connection with the local community [15, 54, 87]. This local product should be seen as another expression of cultural capital that promotes social and economic benefits in rural or peri-urban areas [55, 94], its sensory and symbolic dimensions are also capable of creating a tourist attraction [15, 56].

In turn, urban tourist catering can contribute to an increase in the demand for locally certified produce, and the sustainability of agriculture, rural areas and food systems in general. The catering sector must commit itself to improving its food-system related attitude and operations at all levels, as well as taking an explicit stand on the burning issues of our time, such as waste reduction, improvements in nutrition, food quality and safety and overall sustainability [26].

Fortunately, the trend by restaurants towards a greater use of local food produce is increasing [36]. This is in line with ever-greater numbers of tourists eating in socio-environmentally responsible restaurants that provide a more personal touch and guarantee better nutritional and sensory quality, experiences, price-quality ratio, accessibility and food safety [51]. The place where agri-food raw material originates and is prepared could become an important competitive advantage for catering business organizations and agents as they increasingly try to influence consumers to value products and brands with geographical indications [1, 13].

Restaurants themselves are responsible for promoting local agri-food produce, and its distinctive quality, through direct communication and experience with their clients [15, 47, 55]. One function of the menu is to transmit the restaurant’s image and identify traits, as if it were a letter of introduction [53, 77]. This aims to cause an individual sensorial perception in clients and influence their experience [105], as well as make the restaurant’s socio-environmental responsibility clear [51], providing food biodiversity includes both locally cultivated and wild food species [78], provision with important benefits in terms of production, image, perceived service quality and increased sales [53].

That said it is clear that an effective inter-relation and collaboration between all those involved in the chain of production and sale of local agri-food may significantly increase the potential of small businesses in the catering sector to contribute to territorial development through a strengthening of commercial transactions between suppliers and restaurants.

## Research methods

This research examines the opinions and perceptions of suppliers, chefs and restaurant managers regarding their inter-relations and the commercial and professional dynamics in the local food produce sector. The study has employed a qualitative methodology with the aim of better understanding this phenomenon through the perceptions, meanings, and attitudes explained by the individuals or groups involved, and thus infer its interpretive and theoretical structure [61].

Most studies of restaurants and gastronomy tourism have focused on analysing demand (tourist-consumers) and supply (chefs and restaurant managers), and have paid very little attention to other agents, such as suppliers, who also play an important role in the sector’s value chain. A total of 25 people were initially selected based on their knowledge of the sector, notable position in the commercial and supply network, and/or the recognition awarded to their professional work in catering and gastronomy. Of these, 17 accepted. Participants were emailed and completed a semi-structured self-administered questionnaire of 16 open and closed questions in the case of restaurants, while suppliers answered 20 questions. Table 1 shows the subject blocks, as well as the corresponding items and indicators.

**Table 1:** Questionnaire and qualitative research indicators

Main subject blocks (Suppliers)	Indicators
Agri-food produce supplied to restaurants	Local origin, production types, artisanal/traditional qualities, material and immaterial values
Product quality certificates or awards	Designations of origin, geographical indicators, etc
Restaurants supplied	Commercial types and orientation, consumption of local, certified produce, level of gastronomic skill
Opinions on their current commercial attitude	Sales’ volume, potentialities and demand for local products, future perspectives, professional and personal relations with restaurants, degree of collaboration
Type of restaurant demand of agri-food products	Products, inclusion in menus, seasonality of sales and produce etc

Main subject blocks (Restaurants)	Indicators
Agri-food produce used	Origin, types, qualities, quality certificates or awards, singularities and characteristics, influence of demand on choice
Suppliers normally used	Types, business structure, wholesaler, retailer, commercial and professional relations, degree of collaboration
Menus and dishes	Quality and variety, characteristics, inclusion of traditional products and recipes, prices, behaviour of demand in consumption, type of cuisine, material and immaterial values
Commercial activity	Current volume and perspectives, incidence in local development of gastronomic tourism. Difficulties and problem

*Source*: Authors’ own data

Six suppliers took part in the study. This figure is both significant and sufficient, as we have discovered that the food supply chain in Córdoba is very horizontal, with very few suppliers supplying the vast majority of tourist-oriented restaurants. Some 50% of the suppliers interviewed supply over a hundred establishments in Córdoba; 16.67% supply between 41 and 60 establishments and 33.33% supply 20 establishments. This indicates that the suppliers have a clearly advantageous commercial position when dealing with the city’s restaurants. Eleven participants completed the questionnaire designed for chefs and restaurant managers, and theoretical saturation was used as the criterion for determining the final sample [91].

With the questionnaire, we adopted one of the most common primary-data collection methods, which is often used in qualitative studies. As noted by Denzin and Lincoln [20], such data are more useful when supported and justified by qualitative information, we thus deemed it necessary to contact the restaurant and supply professionals, as they were those best placed to provide first-hand opinions on what may prove decisive in providing gastronomic products and services that satisfy the tourist.

Triangulation was used for data analysis, contrasting a range of information sources, opinions, and focuses to ensure the overall reliability of the study [60]. Data sources came from the questionnaires distributed, information gathered from menus, the restaurants’ official websites and digital marketing. This technique has permitted a more concise, complete analysis of the data, which are more consistently ordered and systemized [60] through the choice of an unintentional convenience sample of those respondents.

**Fig. 1:**
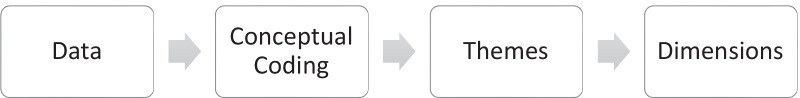
Data analysis process. *Source*: Authors’ own data

Each of the questionnaires was read in full, and information extracted and noted throughout, as well as notes made on reflection as the reading process continued. The quantitative data collected were tabulated and representative graphics were generated using the MsExcel and the qualitative data were treated using NVIVO12 software for data coding and descriptive analysis. Once data were compiled, the survey protocol and corresponding codes/nodes were designed (Fig. 1). The data codes/nodes were classified under five main headings: agri-food and raw materials; supply chain; commercial relations between suppliers and restaurants; gastronomic cultural heritage; and actions taken in response to the crisis caused by COVID-19.

Furthermore, a documentary review was carried out [61] to gather information regarding company structure, sector characteristics, inclusion of local products, references to suppliers and traditional dishes on the menus of those restaurants.

## Findings and discussion

The content analysis identified four main themes, and several subthemes, that have been used to organise and present de following section.

### Agri-food produce and raw materials

The suppliers stated that the vast majority of their sales were of artisanal produce, while sales of industrially processed food were minimal. Some 50% of the suppliers classified their production as ‘local’, the other 50% preferred to use the backing of quality certificates, among them Designation of Origin, and Geographical Indications.

A total of 37.5% of the agri-food suppliers to the city is catering establishments offered Designation of Origin certified produce. Of these, 25% were products derived from Iberian pork, and 12.5% wines; ham and wine are strongly rooted products in the food culture and heritage of local Cordoban districts. The remaining suppliers (25%) provided cheese and meats, and 12.5% were ecologically produced local fruit and vegetables, and dairy products, all in similar proportions.

According to the chefs and restaurant managers, the criteria used when choosing suppliers were: (1) having P.D.O. certified 19.44%); (2) having traditionally processed raw material originating in the Córdoba area (16.67%); (3) speedy and functional delivery (16.67%); (4) the supplier’s head offices being located in Córdoba province (13.89%); (5) the availability of products that other suppliers lacked (11.12%); and (6) that suppliers had food quality, commercial, or environmental certificates (8.33%). It should be highlighted that 40% of the restaurants only work with certified companies, and that certification plays an important role in the decisions of the remaining 60%.

Restaurant menus are fundamental in communication with the client [53, 77, 105]. Those examined in this study show that 80% make express reference to and promotion of P.D.O. ingredients,70% refer to the general quality of ingredients, their seasonal nature, and special flavours; and a further 60% state the geographical origin of ingredients as being from the province of Córdoba. We can thus confirm that the city’s tourist restaurants are adapting to trends and changing habits of new kinds of clients, who are increasingly interested in knowing just where the food on their plates comes from [47]. They are also interested in taste, texture, freshness, food safety, traditional production methods [33], and overall quality as differentiating values in a market that is making increasingly competitive use of certificates of quality and marketing seals [1]. This competitiveness is an opportunity to guarantee the sustainability of the local territory and promote local culture based on the identity of unique food products [89].

### Supply chain

As detailed above, the supply chain in Cordoba is exceptionally horizontal, few suppliers work with many restaurants. Córdoba’s tourist restaurants are concentrated in the most important historic areas, some 40% are found in the Judería and San Basilio neighbourhoods, where some of the city’s most important tourist sites can be found. Indeed, suppliers stated that just 27.5% of their production is distributed in the newly built northern and eastern suburbs, where restaurants specialize more in avant-garde and international cooking, alongside more typical establishments and fast-food outlets. The less touristy areas of the centre, have few tourist restaurants, their offer being aimed more at local clientele—they account for just 20% of the suppliers’ produce; and a mere 12.5% is distributed to establishments in areas to the north and west of the centre, and on the city’s outskirts.

The range of products supplied is wide and varied, taking in practically all possible market segments. Some 21.43% of products are supplied to restaurants classified as traditional and Mediterranean; 17.86% to creative and/or avant-garde restaurants; the same percentage is supplied to establishments with a clear focus on tourists; 14.29% is supplied to traditional taverns; 10.71% goes to gourmet markets; another 10.71% is supplied to local non-tourist restaurants; and 7.14% goes to big name franchise establishments.

The research also showed that 71.43% of suppliers’ food and/or agri-food is distributed in the city of Córdoba, while the remaining 28.57% is distributed in the rural areas of the province. If we consider the distance and origin of the produce, then 50% are local, provincial or regional products. Some 12.5% are produced within 50 km of the city, and 37.5% are produced less than 200 km away. Nonetheless, there is a wide offer of similar produce from other areas of Spain (37.5%) and other countries (12.5%), the majority of the latter being from European Mediterranean countries. The chefs and restaurant managers consulted corroborated these data. The predominance of local produce in the city’s tourist restaurants is, therefore, in line with the recommendations of the United Nations’ 2030 Agenda for Sustainable Development by promoting the consumption and sustainable production in the area of gastronomic tourism [96].

It is worth noting that the vast majority of chefs consulted in the study remarked that the use of local ingredients was essential to their cooking. They all felt that the quality of such produce directly influences the authentic, genuine nature of the dishes they serve to their clients, and that they are fundamental in providing clients with satisfactory experiences perceived through tastings, as well as the aesthetics and presentation of the dishes themselves [14, 53]. The restaurants surveyed are ever more likely to promote the new hyperlocal food trend [19].

A closer look at these results leads us to note how the suppliers declared that just 20% of the demand of Córdoba’s tourist restaurants is based on price-quality ratio. For the remaining 80%, key factors are proximity of production, product quality, ecologically produced and certified. The supply of such produce is linked to trust in producers and their professional trajectory, and they are carefully selected to ensure the desired quality of service. In line with this, the interviewed suppliers stated that they try to personalize their deliveries, looking for specific, differentiating characteristics, and high quality for the city’s tourist restaurants.

Some 66.67% of suppliers perceive that the restaurants change their menus to include seasonal produce. Restaurants permit suppliers a degree of flexibility when offering higher quality produce at a better price depending on the conditions at any specific time [45]. Based on the restaurant survey and the study of menus, we note that, while menus are more or less unchanging year-round, with a mixture of traditional and more innovative dishes, the ingredients used may undergo some seasonal change. These changes also contribute to biodiversity by respecting the natural cycles of agricultural production and minimizing the food waste so many restaurants across the continent are responsible for [12, 19, 78]. Furthermore, the use of gastronomic produce and resources by chefs contributes to the sustainability of the gastronomic tourism value chain by stimulating their creative process, diversifying their menus, continually innovating their cuisine and surprising their regular clients [14, 22].

It should be noted that the suppliers see and value their activity as an instrument for developing the local economy, and thereby helping local and provincial producers. A huge 90% of the restaurants surveyed felt the same regarding their contribution to the production of added value in the creation of jobs, and income, and improving the quality of the gastronomic tourism on offer.

Tourist activity in the city is, in any case, highly seasonal, with high season running from April to June. This has a direct impact on restaurants; 66.68% of suppliers said that the consumption and supply of their produce increases exponentially during high season, which is a key period that offers the best conditions for establishing new commercial relations. Nonetheless, while both restaurants and suppliers identified a seasonal change in the quantity of products available, the same was not true of their price. This is evidence that suppliers are aware of the need for the same levels of quality all year round.

### Commercial relations

Over 65% of the suppliers considered relations with restaurants to be fluid and highly-personalized. These relations take place in a context of cordial dialogue that generally occurs on visits to restaurants, promotional activities, technical visits to production sites, or supplier-promoted gastronomic tastings. Some 83% of suppliers stated that such activities foment a collaborative atmosphere of mutual trust, indicating that the actual purchase is made by the restaurant owner/manager or chef. This collaboration has a positive impact on the supply chain and the performance of suppliers [2, 81, 104], as it facilitates the exchange of experiences, better treatment, transformation and preparation of ingredients, strengthening the value chain through cooperation and boosting local production. Some restaurants even stated that these special relations could lead to the synchronization of activities, decision-making and the exchange of relevant food-related resources and information [18, 104].

Effective integration between suppliers and purchasers is seen to be a key strategy in good management and improving restaurants’ financial performance; in the long term, this may lead to greater efficiency in problem-solving and daily decision-making [18]. Some of the restaurants surveyed have at times realized that commercial relations with suppliers have been too conventional, hindering adaptation to operations that call for specific, unusual or high-quality ingredients. Therefore, they have realized that establishing more personalized relations is vital in achieving a satisfactory solution, guaranteeing a good price-quality ratio, product quality and medium- and long-term guaranteed supply.

From the suppliers’ perspective, the traditional gastronomic sector in Córdoba has been somewhat slow to recognize the importance of product quality and the value of local production. This is no longer the case, with highly recognized restaurants being the reason for this. Some 50% of suppliers stated that such restaurants are professional and seek to improve their distinctiveness and competitiveness, while 33% sustained that the notable increase in demand over recent years has led some restaurants to show little interest in improving their professional quality. Such opinions may well change in light of the current COVID-19 crisis, which will demand added efforts in terms of sustainability and improved client service to remain competitive in a market that will recover only over time and with great difficulty.

The suppliers stated that they must generate a number of externalities for restaurants in the historic centre and the area of greatest tourist movement, the costs of which have to be assumed in the market price. Another complication arises from the nature of the historical centre. It is one of the largest in Europe [101], and its narrow streets and time restrictions on traffic make vehicle access very difficult, meaning that deliveries take longer.

The lack of regulation or agreements related to price leads to a large number of suppliers competing with far lower prices, and consequently quality is lower too. It is in this context that relations between supplier and restaurant come into play once more; the personal relationship and trust that have been built up over time works to the advantage of those suppliers whose offer is based on quality rather than price.

### Food culture heritage

The suppliers of agri-food produce acknowledged the immaterial value of their products [46]. They mentioned concepts such as wisdom, collective memory and local, endogenous traditions. Some 83% of those indicated that they considered and valued not only material aspects, such as freshness, taste and hygiene, but also immaterial ones such as origin, culture and tradition.

Notable among the most highly-valued immaterial aspects are the means of production, the cultural value of the traditional activity, the popular wisdom of ecological smallholders, the systems of cooperative and collaborative work that still exist, the contribution of local producers to conserving methods, processes, and ways to preserve tastes and ancestral wisdom, which together generate high-quality, exclusive products through a production and transformation process that is eminently artisanal.

From the restaurants’ standpoint, some 90% recognized that their cuisine valued local gastronomic culture and collective memory, ancestral traditions and cultural context. This is reflected in immaterial values such as the artisanal process, authenticity, the wisdom of traditional cooking; all of these, if well managed and promoted, can provide a unique gastronomic experience that is intrinsically linked to the qualities of the local surroundings. The higher value given to immaterial gastronomic aspects that this research has noted is a sign of an emerging trend towards solidarity and empathy between consumers, restaurants, and producers. A tourist destination thus becomes an authentic “stage”, where alternative spaces and “rural” provincial life take shape and support one another in the face of current problems and concerns regarding rising costs, low profitability, new challenges and the scarce social recognition of rural professions.

This social interaction that takes place through catering and gastronomy between tourists and producers is certainly important in awarding meaning and value to the concept of ‘*terroir’*. This is transmitted to consumers through traditional Cordoban cuisine, and the images of a local rural production that is far removed from “non-traditional” methods of artificial standardization and regulation. The connection between producers/consumers through gastronomic tourism improves the tourist and cultural image of the place visited.

The three main dishes with greatest tradition and highest demand on menus are all of part of the city’s historic and cultural tradition. They are Cordoban *salmorejo, flamenquín,* and bull’s-tail stew, although other dishes are also mentioned. Some 80% of menus promote traditional Cordoban dishes, while 50% stress the creation and innovation of their dishes; 30% mention the publicity that comes from the awards and distinctions received by their chefs; and a further 30% highlight the publication and dissemination of their signature recipes.

**Fig. 2:**
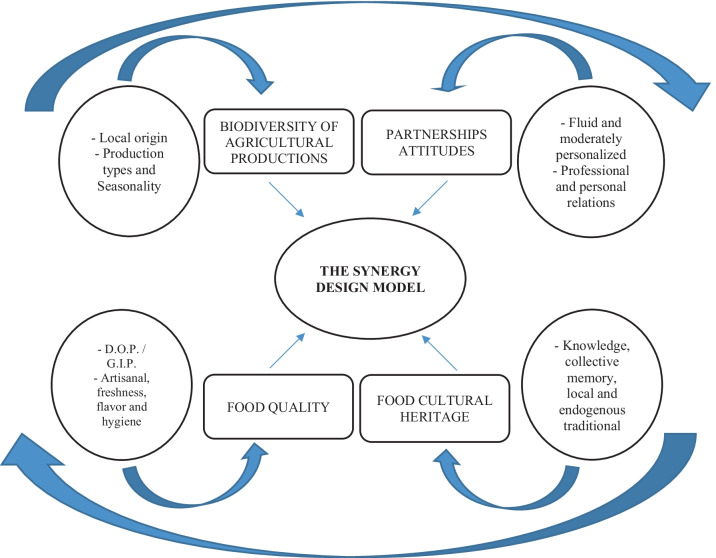
Sustainable model of synergy between food and agri-food suppliers and the restaurant sector in World Heritage City of Córdoba (Spain). *Source*: Authors’ own data

As explained, the analysis of the results obtained has allowed us to identify a series of variables and main dimensions of the central theme that we study in which there are perceptions and visions shared by the suppliers and that also make up the main structure of the value chain of the tourist restoration (Fig. 2).

### Actions undertaken within the context of the COVID-19 crisis

To minimize the negative impact of the loss of restaurant clients, 67% of the suppliers interviewed have adopted new commercial strategies to promote their products directly to the final consumer [44] and in retail food shops, stressing the quality and difference of their products and services and their commitment to the final consumers. They have implemented actions focused on the product, service, communication and commitment to the customer. Produce is now packed and distributed in small quantities,attractively-priced baskets and kits offering a choice of products have been designed; marketing campaigns are now digital and via social networks (promotional videos, interactive chats with gastronomic experts, virtual conferences and cookery classes), referring to the traditional production and elaboration of the food, its geographical origin and recipes. Some suppliers have launched campaigns—such as *#quedateencasa* (#stayhome)—that are designed to involve the consumer in cooking; there has even been a tapas photography competition.

The suppliers have also had to adapt their distribution logistics, promoting home delivery, local pick-up points, free delivery and even one-day delivery for telephone and email orders, along with special discounts.

While the effects of the Covid19 crisis have clearly been significant and caused great uncertainty, such actions show that the sector is dynamic, tries to adapt quickly and flexibly in line with social, economic and cultural changes, and can respond in exceptional situations. The suppliers themselves recognize that, while the pandemic has forced them to face new challenges and problems, it may also be the source of new commercial opportunities.

It remains to be seen whether the integration and generally favourable synergies between suppliers and restaurants that we have identified in this study results in a quick and efficient post Covid19 recovery of gastronomic tourism once restaurants have reopened and safety measures and restrictions on capacity have been eased or lifted and demand slowly increases. These are questions that future research will have to answer, but the first impressions, and the proven resilience of gastronomic tourism, lead us to believe that the sector is capable of responding to some degree to changing demand in times of crisis.

### Considerations

Recognizing the important development potential of Cordoban gastronomy, we have shown that the value that suppliers and tourist restaurants place on the material and immaterial aspects of their traditional cuisine are clear strengths in any gastronomic tourist destination that aims for excellence [82].

One conclusion is that the suppliers, through the raw materials and foods they supply, have also become leading agents in Cordoban cuisine. This finding is new, as most previous research has ignored the role of agri-food suppliers. Their activities bring value to the tradition and quality of local agri-food, and it is largely due to their efforts that restaurants can provide their clients with broader gastronomic experiences, connecting the client with the uniqueness of the local food heritage culture.

This research has provided an understanding of the relations and synergies existing between agri-food suppliers and restaurants. Lastly, our findings permit us to define the market as semi-structured, and multi-polar, with a predominance of traditional suppliers whose activities are very much in line with the specific characteristics of the market and whose professional trajectories have been similar to those of the restaurants they supply.

The tourist catering subsector in the city of Córdoba, as reflected by restaurant chefs and owners/managers, maintains a very direct, close link with the suppliers, which contributes to an exchange of information, experiences, and good practices regarding the handling of agri-food, and the preparation of seasonal dishes and menus. Both groups can certainly benefit from the strengths of local and regional gastronomy, while this, in turn, improves their competitive position in the market.

The fruitful synergy existing between suppliers and restaurants is important when identifying operational and distribution problems and difficulties, wherever they may arise. Consequently, the general results obtained in this case study reinforce the thesis regarding the importance of identifying and understanding the relations between those agents who form part of the value chain of gastronomic tourism.

Although the city of Córdoba has a highly-diversified, representative, and excellent gastronomic offer, changes and improvements still need to be made in restaurant management, the supply of foodstuffs, and the public sector’s running of gastronomic tourism through publicity, commercialization, the design of products and attractions, as well as providing restaurants with support. Examples of such support would be making improvements that facilitate the delivery of food in the historical centre, building nearby car parks, better regulation of supplies, and the design and implementation of a tourist restaurant plan, something the city currently lacks.

In general terms, Cordoba gastronomy has known how to extract value from its traditional dishes and those ingredients most-closely linked with the identity and distinctive nature of the Mediterranean diet. There is now a clear perception of quality, which has become a highly attractive resource in the tourist imaginary of a city that can boast a rich culinary heritage. Consequently, gastronomic tourism has become one of the segments that provides the tourist with greatest satisfaction, and makes them more likely to return. It makes a vital contribution to increasing the richness and diversity of the city’s supply of tourism products and services and increases the competitiveness of tourist companies in general, and restaurants in particular.

Even despite the complex and uncertain context of the Covid-19 pandemic and the months-long closure of most of the city’s restaurants, agri-food suppliers have been able to reinvent themselves and find new market opportunities. They have made the right choices when adapting to markets they had hardly explored beforehand, such as direct sales to a public that is aware of and open to high-quality ingredients. It seems clear that these suppliers will play a vital role as restaurants reopen, restrictions are lifted and national and international tourism recovers in the post-pandemic era.

## Research limitations and future research

We are aware that the study has some limitations. These are principally related to the relatively short research period (three months). This was conditioned by its link to a scientific research and exchange scholarship between two Spanish and Brazilian universities, which was funded by the Fundación Carolina (Spain). We believe that future research should use a larger number of in-depth interviews with a greater number of suppliers. We have observed the importance of these groups in the operational processes with restaurants of agri-food produce commercialization and storage and in the development of gastronomic tourism itself. Longer-term monitoring would be interesting, following recent developments in the literature, as would a study comparing other locations and destinations for such tourism; this would provide even more wide-ranging and significant results and conclusions.

## Data Availability

The dataset used and analysed during the current study is available from the corresponding author on reasonable request.
